# Perioperative Anaesthetic Management and Outcomes in Bariatric Surgery: A Sevenyear Retrospective Cohort Study at Hospital Pakar Universiti Sains Malaysia

**DOI:** 10.21315/mjms-03-2025-193

**Published:** 2025-08-30

**Authors:** Kavitha Kandasamy, Laila Ab Mukmin, Sanihah Che Omar, W Mohd Nazaruddin W Hassan, Umairah Esa, Mohd Nizam Md Hashim, Zaidi Zakaria, Wan Fadzlina Wan Muhd Shukeri

**Affiliations:** 1 Department of Anaesthesiology and Intensive Care, School of Medical Sciences, Universiti Sains Malaysia, Health Campus, Kelantan, Malaysia; 2 Department of Surgery, School of Medical Sciences, Universiti Sains Malaysia, Health Campus, Kelantan, Malaysia

**Keywords:** bariatric surgery, perioperative care, outcome assessment

## Abstract

**Background:**

Perioperative anaesthetic management in bariatric surgery presents unique challenges due to patient comorbidities and elevated perioperative risk. This study aimed to evaluate perioperative anaesthetic practices and determine factors associated with postoperative high-dependency unit (HDU) admissions among bariatric surgery patients at a tertiary centre in Malaysia.

**Methods:**

A retrospective cohort study was conducted involving 104 adult patients who underwent elective bariatric surgery between 2016 and 2022 at Hospital Pakar Universiti Sains Malaysia. Demographic, clinical, surgical, and anaesthetic data were collected. Univariate and multivariate logistic regression analyses were performed to identify factors associated with postoperative HDU admission.

**Results:**

The mean age of the patients was 42.5 years (standard deviation [SD] = 9.7), with a female predominance (68.3%) and a mean body mass index (BMI) of 49.6 kg/m^2^ (SD = 11.3). Obstructive sleep apnoea (OSA) (56.7%) was the most common comorbidity. Laparoscopic sleeve gastrectomy was the most frequently performed procedure (66.3%). The overall HDU admission rate was 34.6%, with a decreasing trend from 2020 to 2022. Patients admitted to the HDU had a significantly higher BMI, more comorbidities, longer operative times, and a higher prevalence of OSA. Multivariate analysis identified OSA (adjusted odds ratio [OR] = 2.6; 95% confidence interval [CI]: 1.003–6.781; *P* = 0.049) and longer duration of surgery (adjusted OR = 2.1; 95% CI: 1.018–4.375; *P* = 0.045) as independent predictors of HDU admission. There were no cases of difficult intubation, postoperative pneumonia, or in-hospital mortality.

**Conclusion:**

Obesity-related diseases, particularly OSA, were highly prevalent. The incidence of difficult intubation was negligible with the use of video laryngoscopy in the ramped position. The HDU admission rates were higher than international benchmarks, influenced primarily by BMI, comorbidities, and surgery duration.

## Introduction

The global rise in obesity poses a growing challenge to healthcare systems, contributing to an escalating burden of comorbidities such as type 2 diabetes mellitus, cardiovascular disease, and obstructive sleep apnoea (OSA) (1). In Southeast Asia, including Malaysia, population-based studies report high obesity prevalence and significant associated comorbid risks (2).

Bariatric surgery offers a durable solution for severe obesity, achieving sustained weight loss and marked improvement in comorbid conditions (2). Enhanced Recovery After Surgery (ERAS) protocols, updated by the ERAS Society in 2021, emphasise multimodal analgesia, early mobilisation, and goal-directed fluid therapy; these have led to reduced postoperative complications and shorter hospital stays among bariatric patients, as demonstrated in both guidelines and randomised trials (3, 4).

Despite these international advancements, anaesthetic management for bariatric patients remains inconsistent. Malaysia still lacks a comprehensive national registry or standard guidelines to evaluate perioperative approaches, including critical outcomes like postoperative high-dependency unit (HDU) admission (5). Although recent institutional reports exist, they cover limited periods or small cohorts (6), leaving nationwide evidence gaps.

This retrospective cohort study reviews seven years of bariatric surgical anaesthesia practices at Hospital Pakar Universiti Sains Malaysia, focusing on perioperative techniques, clinical outcomes, and predictors of postoperative HDU admission. By contributing local data and benchmarking against international ERAS standards, this study’s findings can inform evidence-based perioperative protocols and resource allocation across Malaysian healthcare settings.

## Methods

### Study Design and Patient Selection

This retrospective cohort study was conducted at Hospital Pakar Universiti Sains Malaysia, a university-affiliated tertiary referral centre located in Kelantan, Malaysia. The hospital serves as a major referral centre in the east coast region and is among the few facilities in the country that offer bariatric surgery. Patients were managed according to the Enhanced Recovery After Bariatric Surgery (ERABS) protocol (3), adapted from international ERAS Society guidelines and customised to local institutional practices.

The study included all adult patients (aged ≥ 18 years) who underwent elective bariatric surgery at the hospital between 2016 and 2022. Patients with significantly incomplete or missing data were excluded from the analysis.

**Figure 1:**
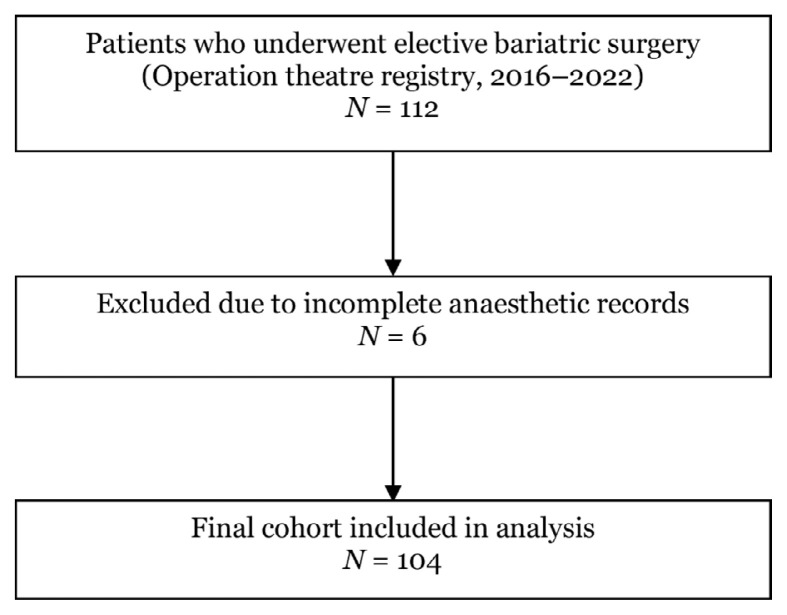
STROBE flow diagram illustrating the selection of patients who underwent bariatric surgery at Hospital Pakar Universiti Sains Malaysia (2016–2022)

A total of 112 patients were identified from the operating theatre registry. After applying the exclusion criteria—specifically, incomplete anaesthetic records (*n* = 8)—a final cohort of 104 patients was included in the analysis. The patient selection process is illustrated in the Strengthening the Reporting of Observational Studies in Epidemiology (STROBE) flow diagram (Figure 1).

### Data Sources

Study data, including baseline demographic, clinical, and surgical characteristics, were initially sourced from the yearly bariatric surgery census. Any missing clinical characteristics were retrieved from medical records, along with perioperative anaesthetic details and outcomes. All data were recorded on manual data collection forms.

Demographic characteristics included age, sex, and body mass index (BMI). Clinical characteristics included the number and type of comorbidities, smoking status, type of bariatric surgery, duration of surgery, intubation technique, arterial line insertion, central venous line insertion, train-of-four monitoring, maintenance of anaesthesia technique, reversal agents, and postoperative analgesic technique. Outcome data included postoperative HDU admission, length of hospital stay (LOS), re-intubation, in-hospital mortality, and postoperative pneumonia.

Postoperative HDU admission was defined as the need for postoperative monitoring and care in the HDU beyond routine post-anaesthesia recovery, based on clinical judgement due to factors such as respiratory compromise, hemodynamic instability, or significant comorbidities. The LOS was measured as the total number of days from the date of surgery to hospital discharge. Re-intubation was defined as the requirement for endotracheal intubation and mechanical ventilation after extubation in the operating room or post-anaesthesia care unit, occurring at any time during the hospital stay. In-hospital mortality was defined as death occurring during the same hospital admission in which the bariatric surgery was performed, regardless of the cause. Postoperative pneumonia was diagnosed based on clinical, radiological, and microbiological criteria, including new or progressive infiltrates on chest imaging, presence of fever (≥ 38°C), leucocytosis or leukopenia, purulent tracheobronchial secretions, and microbiological confirmation if available (e.g., positive sputum culture).

### Bias and Confounding

Data were extracted using a structured data collection form to ensure uniformity and reduce variability in retrieval. To minimise selection bias, all eligible patients who underwent bariatric surgery between 2016 and 2022 were included.

To reduce misclassification bias and missing data, information was cross-checked between the yearly bariatric surgery census and medical records. Any discrepancies were resolved through independent verification by two investigators.

Only complete case analysis was used. Outcomes were recorded based on predefined criteria to maintain consistency across all cases.

### Statistical Analysis

Descriptive statistics were used to summarise baseline demographic and clinical characteristics, perioperative anaesthetic management, and outcomes. Data analysis was performed using Statistical Package for the Social Sciences (SPSS) version 24 (IBM Corp., Armonk, NY, US). Categorical variables are presented as absolute numbers and percentages, while continuous variables are expressed as mean with standard deviation (SD) for normally distributed data, or median with interquartile range (IQR) for non-normally distributed data.

Factors associated with HDU admission were initially explored using univariate logistic regression, with results reported as odds ratios (OR) and 95% confidence intervals (CI). Variables with a *P*-value of < 0.10 in univariate analysis were subsequently included in a multivariate logistic regression model to identify independent predictors of HDU admission. A *P*-value of < 0.05 was considered statistically significant.

### Sample Size Calculation

Due to the retrospective nature of the study, all eligible patients who underwent elective bariatric surgery at the institution between 2016 and 2022 were included. Therefore, a formal sample size calculation was not applicable.

## Results

### Demographic and Clinical Characteristics

The baseline demographic and clinical characteristics of the patients are summarised in Table 1. The mean age of the cohort was 42.5 years (SD = 9.7), with a female predominance (68.3%). The mean BMI was 49.6 kg/m^2^ (SD = 11.3). Sixteen patients (15.4%) had no comorbidities, while the remaining patients had at least one. The most prevalent comorbidity was OSA, affecting 56.7% of patients, followed by hypertension (39.4%) and diabetes mellitus (32.7%). Only five patients (4.8%) were actively smoking at the time of preanaesthetic assessment.

Regarding surgical procedures, laparoscopic sleeve gastrectomy was the most commonly performed operation (66.3%), while 33.7% of patients underwent laparoscopic gastric bypass. The mean duration of surgery was 108 minutes (SD = 36).

### Perioperative Anaesthetic Management and Outcomes

The perioperative anaesthetic management and outcomes are summarised in Table 2. There were no cases of difficult intubation; all patients were intubated successfully using video laryngoscopy in the ramped position, and none required awake fibreoptic intubation.

Arterial line insertion was performed in 19 patients (18.3%), while central venous access was required in only 3 (2.9%). Neuromuscular monitoring using train-of-four was utilised in 21 patients (20.2%).

For maintenance of anaesthesia, desflurane was the most commonly used agent (79.8%), followed by sevoflurane (13.5%) and total intravenous anaesthesia (TIVA) (6.7%). Most patients (95.2%) received intravenous (IV) sugammadex for reversal of neuromuscular blockade.

In terms of postoperative analgesia, IV nonopioid analgesics were administered in 96.2% of patients, while IV opioid patient-controlled analgesia (PCA) was used in 3.8%.

The mean LOS was 5.9 days (SD = 1.2). One patient (1%) required re-intubation within the first postoperative week due to an anastomotic leak. Importantly, there were no cases of postoperative pneumonia or inhospital mortality.

### Trends in Postoperative HDU Admission in Bariatric Surgery

**Figure 2:**
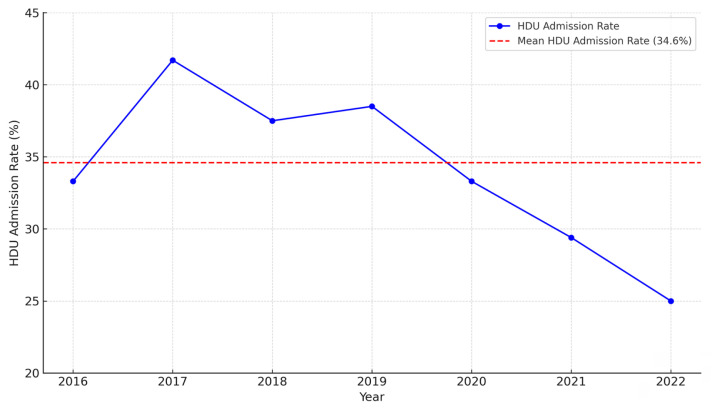
Trends in postoperative HDU admission in bariatric surgery (2016–2022)

Figure 2 shows the annual trend in HDU admission rates among bariatric surgery patients from 2016 to 2022 at Hospital Pakar Universiti Sains Malaysia. The HDU admission rate ranged from 29.4% to 41.7%, with the highest rate observed in 2017 and the lowest in 2021. From 2016 to 2019, the admission rate demonstrated a general upward trend, followed by a gradual decline from 2020 to 2022. The overall mean HDU admission rate for the study period was 34.6%, as indicated by the red dashed line. Notably, all HDU admissions were planned, with no unplanned or emergency transfers.

### Baseline Characteristics of HDU and Non-HDU Admissions

Of the 104 patients who underwent bariatric surgery, 36 (34.6%) were admitted to the HDU postoperatively, while 68 (65.4%) were managed in general wards. Patients in the HDU group had a significantly higher mean BMI compared to the non-HDU group (53.2 vs 47.7 kg/m^2^, *P* = 0.019) (Table 3). The proportion of patients with at least one comorbidity was significantly higher in the HDU group (34/36; 94.4%) compared to the non-HDU group (54/68; 79.4%) (*P* = 0.048). Next, OSA was more prevalent among HDU patients (27/36; 75.0%) than among non- HDU patients (32/68; 47.1%) (*P* = 0.048). Additionally, the mean duration of surgery was longer in the HDU group (118 minutes vs 103 minutes, *P* = 0.037). There were no significant differences between the groups in sex distribution, smoking status, or type of bariatric procedure performed.

### Univariate and Multivariate Analysis of Factors Associated with HDU Admission

The univariate and multivariate analyses of factors associated with postoperative HDU admission are summarised in Table 4. For the univariate analysis, several variables were significantly associated with HDU admission: higher BMI (OR = 1.1; 95% CI: 1.006–1.084; *P* = 0.022), presence of one or more comorbidities (OR = 1.5; 95% CI: 1.047– 2.160; *P* = 0.027), OSA (OR = 3.4; 95% CI: 1.383–8.236; *P* = 0.008), and longer duration of surgery (OR = 2.1; 95% CI: 1.033–4.078; *P* = 0.040).

In the multivariate analysis, two factors remained independently associated with HDU admission: OSA (adjusted OR = 2.6; 95% CI: 1.003–6.781; *P* = 0.049) and duration of surgery (adjusted OR = 2.1; 95% CI: 1.018–4.375; *P* = 0.045). The BMI and the presence of one or more comorbidities did not retain statistical significance after adjustment.

## Discussion

This retrospective cohort study provides important insights into the perioperative anaesthetic management and outcomes of bariatric surgery patients at the centre over seven years. Through the analysis of 104 patients, key trends in demographics, anaesthetic techniques, and postoperative outcomes that contribute meaningfully to the evolving body of knowledge in bariatric anaesthesia were identified.

### Patient Demographics and Clinical Characteristics

Consistent with global patterns, the majority of patients were middle-aged females (mean age: 42.5 years), with a mean BMI of 49.6 kg/m^2^. Severe obesity is notably more prevalent among females seeking bariatric surgery (1), and this study’s findings reflect this epidemiological trend. A substantial proportion (84.6%) had at least one comorbidity, with OSA, hypertension, and type 2 diabetes mellitus being most common. These conditions are well-documented in the bariatric population and present significant anaesthetic challenges (2–6).

The high prevalence of comorbidities such as OSA is particularly relevant due to their association with difficult airway management, altered respiratory mechanics, and increased cardiovascular risk (7). Morgan et al. (8) identified OSA, diabetes, and chronic respiratory diseases as significant predictors of unplanned intensive care unit (ICU) admission following bariatric surgery, underlining the importance of comprehensive preoperative assessment. Notably, in this present study, no major anaesthetic complications were observed despite this high comorbidity burden, suggesting that the centre’s institutional perioperative strategies may effectively mitigate risk.

### Perioperative Anaesthetic Management and Outcomes

The data support a structured and effective approach to anaesthesia in bariatric surgery, with particular emphasis on airway management, anaesthetic maintenance, and postoperative analgesia.

Airway management was universally achieved using video laryngoscopy in the ramped position. This technique addresses the anatomical and physiological challenges of obesity and is consistent with guidelines advocating for advanced airway tools in high-risk patients (9). Importantly, no cases of difficult intubation or the need for awake fibreoptic intubation were reported, challenging the traditional assumption that obesity alone predicts a difficult airway. This observation aligns with the Difficult Airway Society’s 2019 guidance, which advises against routine awake intubation unless specific indications are identified (10).

The selective use of invasive monitoring, such as arterial or central lines, reflects an individualised, risk-based approach. Desflurane was the maintenance agent most frequently used, followed by sevoflurane and TIVA, consistent with contemporary practices aimed at enhancing recovery through rapid emergence and reduced respiratory complications (3, 11). The widespread use of IV sugammadex for neuromuscular blockade reversal further supports this recovery-focused strategy.

Analgesia was predominantly multimodal and opioid-sparing. The limited use of IV PCA reflects effective implementation of enhanced recovery strategies and supports efforts to minimise opioid-related complications—a growing concern in perioperative medicine (12).

The mean hospital stay of 5.9 days is within expected limits for bariatric surgery patients. The absence of in-hospital mortality or postoperative pneumonia further reinforces the safety and efficacy of the perioperative care protocols. Re-intubation was rare (1%) and was attributed to an anastomotic leak, underscoring the importance of vigilant postoperative monitoring and timely intervention when complications arise.

### Trends in Postoperative HDU Admission

The HDU admission rate of 34.6%, with no unplanned admissions, reflects a cautious and proactive perioperative strategy. These figures are comparable to the 36% ICU admission rate reported for an Egyptian cohort (13) but contrast with much lower rates reported by a population-based study from Australia (4.9%) (8). These differences likely reflect variations in institutional protocols, patient selection criteria, and perioperative risk stratification strategies.

The question of whether routine HDU or ICU admission is necessary for all bariatric surgery patients remains debated. While some centres advocate intensive postoperative monitoring of high-risk patients (14, 15), universal admission may not be cost-effective or clinically warranted. The present study’s findings suggest that patient-specific risk stratification is a more appropriate approach. The incorporation of ERAS protocols has been shown to reduce the need for intensive postoperative care while improving recovery (3, 11).

The upward trend in postoperative HDU admission from 2016 to 2019 may reflect heightened caution during the earlier phase of the bariatric surgery programme at the centre, possibly due to limited experience, evolving case complexity, or a lower threshold for HDU admission. During this time, perioperative pathways were still being optimised, and clinicians may have opted for more intensive postoperative monitoring to mitigate perceived risks.

The gradual decline in HDU admissions from 2020 to 2022 likely reflects increased institutional confidence, improvements in surgical and anaesthetic techniques, and the maturation of the bariatric surgery team. Additionally, the more consistent application of ERAS principles—including better patient selection, optimised comorbidity management, and standardised anaesthetic protocols—may have contributed to a reduced need for routine postoperative HDU care (3, 11, 14).

### Factors Determining Postoperative HDU Admission

The findings revealed that higher BMI, the presence of one or more comorbidities, OSA, and prolonged surgical duration were significantly associated with postoperative HDU admission. These factors are well-supported in the literature as predictors of increased physiological burden and risk of postoperative complications (4–6, 16).

Patients with higher BMI often have more complex medical profiles and impaired physiological reserves. When compounded by comorbidities such as cardiovascular disease, diabetes, and respiratory disorders, the perioperative risks are substantially increased. In particular, OSA was significantly associated with HDU admission in this study’s cohort, a finding supported by meta-analyses showing its independent association with adverse postoperative outcomes (17). Finally, prolonged surgery duration, a marker of procedural complexity, may increase intraoperative physiological stress and predispose patients to postoperative instability (18). Identifying these predictors allows clinicians to tailor monitoring and intervention strategies more precisely, ensuring optimal postoperative outcomes.

### Study Limitations

This study has several limitations. First, it was conducted at a single tertiary centre, which may limit the generalisability of the findings to institutions with different patient populations or perioperative protocols. Second, the retrospective design limits the ability to establish causal relationships and is dependent on the accuracy and completeness of documentation. Additionally, although the sample size is comparable to similar studies, it may be underpowered to detect rare complications or to perform more granular subgroup analyses. Future multicentre prospective studies are needed to validate these findings and refine postoperative risk stratification strategies.

### Implications and Future Directions

This study contributes to the regional and global understanding of anaesthetic practices in bariatric surgery. The absence of major anaesthetic complications and the low rate of re-intubation support the feasibility of bariatric procedures without routine ICU admission, provided there is thorough perioperative planning. Future research should focus on prospective validation of HDU risk predictors, cost-effectiveness analyses of selective admission strategies, and the long-term impact of ERAS protocols on recovery and readmission rates.

## Conclusion

This retrospective study highlights that perioperative anaesthetic management of bariatric surgery patients at the centre was effective and safe, with no major anaesthetic complications and a low rate of re-intubation. All postoperative HDU admissions were planned, and their trend over the study period suggests evolving clinical practices and improved perioperative protocols. Higher BMI, the presence of comorbidities, OSA, and lengthier surgical duration were associated with HDU admission. These findings support the selective use of postoperative HDU care based on individual risk assessment. Further prospective multicentre studies are needed to validate the results and optimise perioperative strategies for bariatric surgery patients.

## Figures and Tables

**Table 1:** Baseline demographic and clinical characteristics of patients who underwent bariatric surgery at Hospital Pakar Universiti Sains Malaysia from 2016 to 2022

Variable	Mean (SD) or frequency (%)(*n* = 104)
***Demographic characteristics***	

Age (years)	42.5 (9.7)

Gender	
Female	71 (68.3)
Male	33 (31.7)

BMI (kg/m^2^)	49.6 (11.3)

***Clinical characteristics***	

Comorbidity status	
Nil	16 (15.4)
≥ 1 comorbidity	88 (84.6)

Type of comorbidity	
OSA	59 (56.7)
Hypertension	41 (39.4)
Diabetes mellitus	34 (32.7)
Knee osteoarthritis	33 (31.7)
Bronchial asthma	14 (13.5)
Ischaemic heart disease	3 (2.9)
Congestive cardiac failure	0

Smoking	5 (4.8)

Type of bariatric surgery	
Laparoscopic sleeve gastrectomy	69 (66.3)
Laparoscopic gastric bypass	35 (33.7)

Duration of surgery (min)	108 (36)

**Table 2:** Perioperative anaesthetic management and outcomes of patients who underwent bariatric surgery at Hospital Pakar Universiti Sains Malaysia from 2016 to 2022

Variable	Mean (SD) or frequency (%)(*n* = 104)
***Perioperative anaesthetic management***	

Difficult intubation	0

Intubation techniques	
Video laryngoscopes in ramped position	104 (100)
Awake fibreopticintubations	0 (0)

Arterial line insertion	19 (18.3)

Central venous line insertion	3 (2.9)

Train-of-four monitoring	21 (20.2)

Maintenance of anaesthesia Desflurane	
Sevoflurane	83 (79.8)
TIVA	14 (13.5)
	7 (6.7)

Sugammadex reversal	99 (95.2)

Postoperative analgesic technique	
IV non-opioid analgesia	100 (96.2)
IV patient-controlled opioid analgesia	4 (3.8)

***Postoperative outcomes***	

LOS (days)	5.9 (1.2)

Re-intubation within one week	1 (1.0)

In-hospital mortality	0

Postoperative pneumonia	0

**Table 3:** Baseline demographic and clinical characteristics of patients undergoing bariatric surgery, stratified by HDU admission status

Variable	HDU (*n* = 36)	Non-HDU (*n* = 68)	*P*-value
***Demographic characteristics***			

Age (years)	42.4 (9.5)	42.5 (9.8)	0.990^a^

Gender			0.798^b^
Female	24 (66.7)	47 (69.1)	
Male	12 (33.3)	21 (30.9)	

BMI (kg/m^2^)	53.2 (13.9)	47.7 (9.1)	0.019^a^

***Clinical characteristics***			

Comorbidity status			
Nil	2 (5.6)	14 (20.6)	0.048^b^
≥ 1 comorbidity	34 (94.4)	54 (79.4)	

Type of comorbidity			
OSA	27 (75.0)	32 (47.1)	0.006^b^
Hypertension	14 (38.9)	27 (39.7)	0.935^b^
Diabetes mellitus	14 (38.9)	20 (29.4)	0.327^b^
Knee osteoarthritis	11 (30.6)	22 (32.4)	0.851^b^
Bronchial asthma	7 (19.4)	7 (10.3)	0.193^b^
Ischaemic heart disease	1 (2.8)	2 (2.9)	0.962^b^

Smoking	2 (5.6)	3 (4.4)	0.795^b^

Type of bariatric surgery			0.356^b^
Laparoscopic sleeve gastrectomy	26 (72.2)	43 (63.2)	
Laparoscopic gastric bypass	10 (27.8)	25 (36.8)	

Duration of surgery (min)	118.0 (36.3)	103.2 (34.5)	0.037^a^

a independent *t*-test;

b chi-square test
